# Pyruvate kinase M2 (PKM2) improve symptoms of post‐ischemic stroke depression by activating VEGF to mediate the MAPK/ERK pathway

**DOI:** 10.1002/brb3.2450

**Published:** 2021-12-13

**Authors:** Yun Feng, Xuebin Li, Jie Wang, Xiaohua Huang, Lanqing Meng, Jianmin Huang

**Affiliations:** ^1^ Department of Neurology The Affiliated Hospital of Youjiang Medical University for Nationalities Baise City Guangxi Province China; ^2^ Department of Neurology Youjiang Medical College for Nationalities Baise City Guangxi Province China; ^3^ Department of Nephrology The Affiliated Hospital of Youjiang Medical University for Nationalities Baise City Guangxi Province China

**Keywords:** ERK, MAPK, PKM2, PSD, VEGF

## Abstract

**Purpose:**

To evaluate and identify the effects and explore the mechanisms of pyruvate kinase M2 (PKM2) on stroke‐induced post stroke depression (PSD).

**Methods:**

Rats were separated into six different groups, including sham + saline, Stroke + saline, PSD + saline, PSD + recombinant pyruvate kinase M2 (rPKM2) (112 ng/kg), PSD + rPKM2 (224 ng/kg), and PSD + rPKM2 (224 ng/kg) + bevacizumab. Then, the body weight, sucrose preference rate, immobility time, horizontal movement, and vertical movement were determined to evaluate the effect of PKM2 on improving the depressive behavior of PSD rats. Subsequently, the proliferation of oligodendrocytes in subventricular zone (SVZ) of rats in each group was examined by western blot and immunofluorescent staining. Furthermore, the mRNA and protein expression levels of TNF‐α, IL‐6, and IL‐1β were also detected by qPCR and ELISA to verify the anti‐inflammatory effects of PKM2 on PSD rats. In addition, the protein expression levels of MDA, LDH, and NO were tested to reveal that PKM2 can reduce oxidative stress in PSD rats. The western blot and IHC assays were employed to examine the protein expression levels of VEGF, PKM2, and ERK in PSD rats.

**Results:**

In this study, the results showed that PKM2 can improve the depressive behavior and proliferation of oligodendrocytes in PSD rats. In addition, PKM2 has anti‐inflammatory and anti‐oxidative stress effects on PSD rats. Meanwhile, PKM2 activated the expression level of VEGF/MAPK/ERK pathway.

**Conclusion:**

PKM2 improves symptoms of post‐ischemic stroke depression by activating VEGF‐mediated MAPK/ERK pathway.

## Graphical abstract

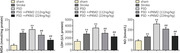

## 1 INTRODUCTION

Post stroke depression (PSD) is a mood disorder characterized by a series of depressive symptoms in stroke patients, which can be manifested as mood swings, sluggishness, irritability or apathy, anhedonia, pessimism, suicidal ideation, and so on (Robinson & Jorge, 2016). As for the pathogenesis of PSD, the current research still does not have a clear mechanism. The occurrence of PSD may be related to stroke induced white matter damage, which leads to the damage of neural circuits or pathways related to emotion regulation (Albert, 2018).

The main manifestation of white matter injury (WMI) is demyelination of myelin sheath. The oligodendrocytes (OLs) can form myelin sheath structures and the oligodendrocyte progenitor cells (OPCs) in the subventricular zone (SVZ) can also differentiate to form myelin sheath, so mature OLs and OPCs are the two most important endogenous sources of myelin repair/regeneration (Jia et al., 2019; Liu et al., 2019). Therefore, preventing OLs from degeneration and death, supplementing enough OLs, and improving OPCs differentiation are one of the main ideas for preventing and treating WMI.

MAPK/ERK signaling pathway also plays an important role in maintaining the long‐term survival of OLs and regulating the fate of OLs differentiation. It can also act on the cell branches of OLs to influence the formation of myelin sheath (Younes‐Rapozo et al., 2009). In addition, phosphorylated ERK signaling pathway can be involved in a variety of biological reactions, such as cell proliferation and differentiation, cytoskeleton construction, cell apoptosis, and inflammatory response (Zohrabian et al., 2009). It has been reported that activation of MAPK‐ERK‐CREB pathway by agonists can reduce the apoptosis and inflammation of renal tubule cells mediated by hypoxia‐reoxygenation injury, thereby alleviating the impact of renal injury (Dong et al., 2019). Curcumin can treat atherosclerosis by reducing inflammatory factors and oxidative stress factors in macrophages through the ERK‐dependent pathway (Ouyang et al., 2019).

Pyruvate kinase M2 (PKM2) is a member of the four pyruvate kinase isoforms (PKM1, PKM2, PKL, and PKR) expressed in mammalian cells (Zhang et al., 2019). PKM2 is highly expressed in proliferating cells, such as cancer cells (Bluemlein et al., 2011; Desai et al., 2014). Expression and activity of PKM2 are regulated at multiple levels, including gene expression, selective splicing, and post‐translational modification, as well as metabolic intermediates and growth signaling pathways (Gao et al., 2013). Thus, PKM2 is a unique multifaceted regulator that can improve cell adaptation in its metabolic state to adapt to physiological needs in different environments (Dong et al., 2019). PKM2 has been reported to mediate angiogenesis, neuroprotection, and functional recovery in adult mouse models of ischemic stroke by increasing STAT3 and focal adhesion kinase activity (Chen et al., 2018). PKM2 also regulates the NRF2 pathway, thereby inhibiting benzoquinone induced oxidative stress in yolk sac hematopoietic stem cells (Zhu et al., 2014). The increased expression of PKM2 by neutrophils at the wound site can promote early wound healing through promoting angiogenesis (Zhang et al., 2016).

Vascular epithelial growth factor (VEGF) has the effect on angiogenesis, and MAPK/ERK is the downstream target of VEGF. Recent studies have found that VEGF plays a role in brain injury repair and nerve nutrition in the central nervous system and can promote the proliferation and differentiation of neural stem cells, neural precursor cells, and even glial cells. Chen et al. found that PKM2 can activate the expression of VEGF, thereby mediating angiogenesis in the mouse model of stroke and improving the effect of stroke (Chen et al., 2018). However, PKM2 and VEGF have been rarely reported in PSD, and the mechanism of OLs regeneration is not clear.

In this study, it was found that PKM2‐mediated MAPK/ERK pathway activity through the activation of VEGF and improved post‐ischemic stroke depression.

## 2 MATERIALS AND METHODS

### 2.1 Animals and establishment of Stroke or PSD rat model

Eight‐week‐old male Sprague–Dawley rats (245‐275 g) were obtained from BIORAY LABORATORIES Inc. (Shanghai, China). All experimental procedures and animal welfare were carried out in accordance with the National Institutes of Health Laboratory Animal Care and Use Guidelines (National Research Council Committee for the Update of the Guide for the & Use of Laboratory, 2011), and the protocols used were approved by the Animal Ethics Committee of the Affiliated Hospital of Youjiang Medical University for Nationalities.

Thirty‐six Sprague–Dawley rats were separated into six different groups (*n* = 6) at random, including sham + saline, Stroke + saline, PSD + saline, PSD + rPKM2 (112 ng/kg), PSD + rPKM2 (224 ng/kg), and PSD + rPKM2 (224 ng/kg) + bevacizumab. Rats in the stroke treated groups underwent ketamine/xylazine (ketamine 80−100 mg/kg i.p., xylazine 10−12.5 mg/kg i.p.) anesthesia and surgery to cause stroke by middle cerebral artery occlusion (MCAO) according to the protocol (Lowrance et al., 2015) and rats in the PSD treated groups underwent surgery to cause PSD according to the protocol (Wang et al., 2019). Briefly, the rats in the PSD group were exposed to chronic unpredictable mild stress treatment, which included electric shock to the foot, water deprivation, food deprivation, tail clamping, and behavioral restriction.

### 2.2 Drug administration

On the first day after PSD establishment, rats in PSD + rPKM2 (112 ng/kg) and PSD + rPKM2 (224 ng/kg) groups received rPKM2 administration, and rats in sham + saline, Stroke + saline, and PSD + saline groups received equal volume of 0.9% saline. The rPKM2 was obtained from Zhiren Liu's group (Li et al., 2014; Zhang et al., 2016) and rPKM2 or saline was administered intranasally every day.

### 2.3 Body weight measurements and animal behavioral tests

Body weight measurements and behavioral experiments were performed 22 days after the rats were administrated with drug.

Sucrose preference test (SPT): On the first day, the rats were given free access to two bottles with 200 mL 1% sucrose solution. The next day, one of the bottles was replaced with fresh water. On the third day, the rats were deprived of food and water for 22 h, after which they were given free access to a weighing bottle containing 200 mL 1% sucrose solution and a weighing bottle containing fresh water. After 1 h, the two bottles were switched, and after another hour, the two bottles were weighed. Sucrose preference (%) is calculated as sucrose consumption (sucrose consumption + water consumption) * 100%.

Forced swimming test (FST): The rats were placed in a transparent pool and their swimming time was recorded with a digital camera for 6 min, and the time when the rats stopped struggling within last 5 min was recorded.

Open field test (OFT): The rats were placed in a test box that had been cleaned with 75% alcohol and recorded free movement for 10 min. The time of immobility of the central area (central occupation), the frequency of erection (holding the hind paws on the floor), and the incidence of modification (including cleaning of the forelimbs, hind paws, face, body, and genitals) were recorded and analyzed.

### 2.4 Western blot

The brain tissues from the subventricular zone (SVZ) or serum samples were collected at 14 days after stroke. Briefly, tissues were washed in pre‐cooling PBS buffer three times, and the total protein was separated by RIPA (Beyotime, Shanghai, China). BCA protein assay kit (CoWin Biotechnology) was applied to detect the protein concentration. An equal amount of total proteins was electrophoresed to SDS‐PAGE. Then, they were transferred to the PVDF membranes (Millipore) with treated by 5% non‐fat milk for 1 h. The protein was identified by incubated with specific primary antibodies Olig‐2 (Rabbit Anti‐Olig‐2 antibody, ab109186, 1:2,500; Abcam, Cambridge, MA, USA), MBP (Rabbit Anti‐MBP antibody, ab218011, 1:3,000; Abcam), TNF‐α (Rabbit Anti‐TNF‐α antibody, ab205587, 1:3,000; Abcam), IL‐6 (Goat Anti‐IL‐6 antibody, AF506, 1:2,500; R&D Systems, Minnesota, USA), IL‐1β (Rabbit Anti‐IL‐1β antibody, ab205924, 1:3,000; Abcam), PKM2 (Rabbit Anti‐PKM2 antibody, ab137852, 1:3,000; Abcam), VEGF (Rabbit Anti‐VEGF antibody, ab46154, 1:2,500; Abcam), p‐ERK (Rabbit Anti‐p‐ERK antibody, ab201015, 1:3,000; Abcam), ERK (Rabbit Anti‐ERK antibody, ab17942, 1:3,000; Abcam), and β‐actin (Rabbit Anti‐beta Actin antibody, ab8227, 1:3,000; Abcam) overnight at 4°C. Then, the membranes were further incubated with HRP‐conjugated goat anti‐rabbit immunoglobulin G secondary antibody (ab205718, 1:2,000; Abcam) and the bands on the membranes were visualized by the ECL chemiluminescence reagent (Beyotime). The analyzed samples were normalized by β‐actin and protein bands were quantified by gray value analysis by ImageJ software (National Institutes of Health).

### 2.5 Immunofluorescent staining

The brain tissues from the subventricular zone (SVZ) or serum samples were collected at 14 days after stroke. Briefly, tissues were fixed by 4% paraformaldehyde and permeabilized by 0.15% Triton X‐100 for 30 min, respectively. Then, cells were blocked by 5% BSA for 1.5 h and incubated with primary antibody Brdu (Rat Anti‐Brdu antibody, ab6326, 1:1,500; Abcam) and Olig‐2 (Rabbit Anti‐Olig‐2 antibody, ab109186, 1:2,000; Abcam) overnight at 4°C. Finally, cells were incubated with florescent‐dye conjugated secondary antibody (Abcam) for 1.5 h followed by staining with DAPI.

### 2.6 qPCR

The brain tissues from the subventricular zone (SVZ) or serum samples were collected at 14 days after stroke. Trizol reagent (Invitrogen, Grand Island, NY, USA) was applied to extract the total RNA from collected cells. The quantity and integrity of extracted total RNA were evaluated on a Nano Drop 1000 spectrophotometer (Thermo Fisher Scientific, Inc.). The expression of TNF‐α, IL‐6 and IL‐1β were detected through qRT‐PCR by the SYBR Premix EX Taq (Takara, Japan). β‐actin were used as the endogenous reference genes to normalize mRNA expression levels. The relative expression of TNF‐α, IL‐6 and IL‐1β in each experimental group were analyzed by the 2^–△△Ct^ method. All reactions were performed in triplicates. Primer sequences are shown in Table 1.

**TABLE 1:** Primers for TNF‐α, IL‐6 and IL‐1β and reference genes

Gene	Primer	Sequence(5′→3′)
TNF‐α	Forward	GCCACCACGCTCTTCTGTCTAC
	Reverse	GGGTCTGGGCCATAGAACTGAT
IL‐6	Forward	CACATGTTCTCTGGGAAATCG
	Reverse	TTGTATCTCTGGAAGTTTCAGATTGTT
IL‐1β	Forward	ACCTTCCAGGATGAGGACATGA
	Reverse	CTAATGGGAACGTCACACACCA
β‐actin	Forward	GTGACGTTGACATCCGTAAAGA
	Reverse	GCCGGACTCATCGTACTCC

### 2.7 Enzyme‐linked immunosorbent assay

For detecting the MDA (ab100768; Abcam), LDH (ab102526; Abcam), and NO (ab65328; Abcam) protein levels, enzyme‐linked immunosorbent assay (ELISA) kits were used. The expression of MDA, LDH, and NO in the serum of rats were assayed with corresponding ELISA kits in line with the manufacturer's instructions.

### 2.8 Immunohistochemical staining

Formalin‐fixed paraffin‐embedded tissues were sectioned and mounted on slides. After deparaffinization in xylene, rehydration, and antigen retrieval in citrate buffer solution (10 μm), sections were blocked with 3% hydrogen peroxide for 25 min. Then the sections were blocked by 10% BSA and incubated with primary antibodies overnight at 4°C. The sections were subsequently incubated with the avidin‐biotin‐peroxidase complex (Thermo Fisher Scientific, Inc.) for 25 min. The nuclei were counterstained with Mayer's hematoxylin solution (Sigma‐Aldrich, St. Louis, MO, USA).

### 2.9 Statistical analysis

All data are shown as the mean ± standard error of the mean from three independent experiments. *p* Values of < 0.01 (two‐tailed) were considered to indicate a statistically significant difference. GraphPad Prism 5 (GraphPad Software, Inc.,) was used for analysis.

## 3 RESULTS

### 3.1 PKM2 can improve the depressive behavior of PSD rats

**FIGURE 1:**
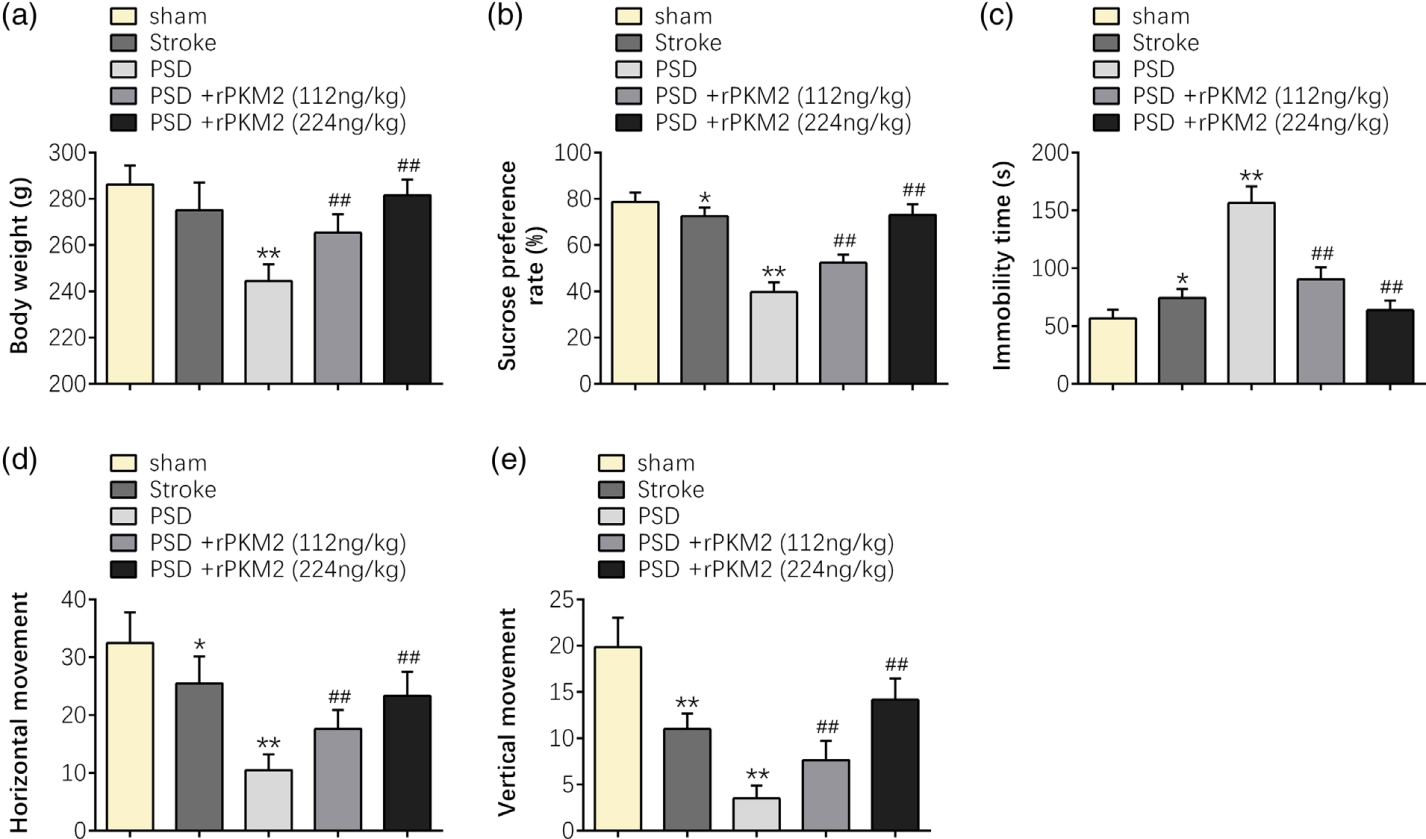
PKM2 can improve the depressive behavior of PSD rats. (a) The body weight. (b) The sucrose preference rate in SPT. (c) The immobility time in FST. (d,e) The horizontal movement and vertical movement in OFT of sham, stroke, or PSD rats at indicated time points in response to intranasal administration of saline or PKM2. Data were presented as the mean ± SD with three independent experiments. *p < .05, **p < .01 versus sham group, and ^##^ *p* < .01 versus PSD group

In this research, the animal stroke disease model was first established to further establish the PSD disease model according to the protocol. Then, the animal behavioral tests were applied to observe the effect of PKM2 on the behavioral performance of rats that underwent PSD surgeries. First, the results revealed that the body weight of the rats was significantly reduced after stroke and PSD surgeries (*p* < 0.01). However, the body weight of the PSD rats was improved by intranasal administration of PKM2 in a dose‐dependent manner, particularly at the concentration of 224 ng/kg (*p* < 0.01) (Figure 1a). Higher body weight indicated that PKM2 displayed improvement effect on the depressive behavior of PSD rats. In addition, sucrose preference test (SPT), forced swimming test (FST), and open field test (OFT) were also applied to observe the effect of PKM2 on the improvement of the depressive behavior of PSD rats. The results indicated that the sucrose preference rate, horizontal movement, and vertical movement of the rats were dramatically reduced, and the immobility time of the rats was strikingly enhanced after stroke and PSD surgeries (*p* < 0.05 or *p* < 0.01). However, the above trends were improved and reversed by intranasal administration of PKM2 in a dose‐dependent manner, particularly at the concentration of 224 ng/kg (*p* < 0.01) (Figure 1b‐e ). The decreased percentage of sucrose intake, more immobility time spent in the central, or less frequency of erection and incidences of modification are the indicators for depressive like symptom. These results suggested that PKM2 can improve the depressive behavior of PSD rats.

### 3.2 PKM2 can promote the proliferation of oligodendrocytes in PSD rats

**FIGURE 2:**
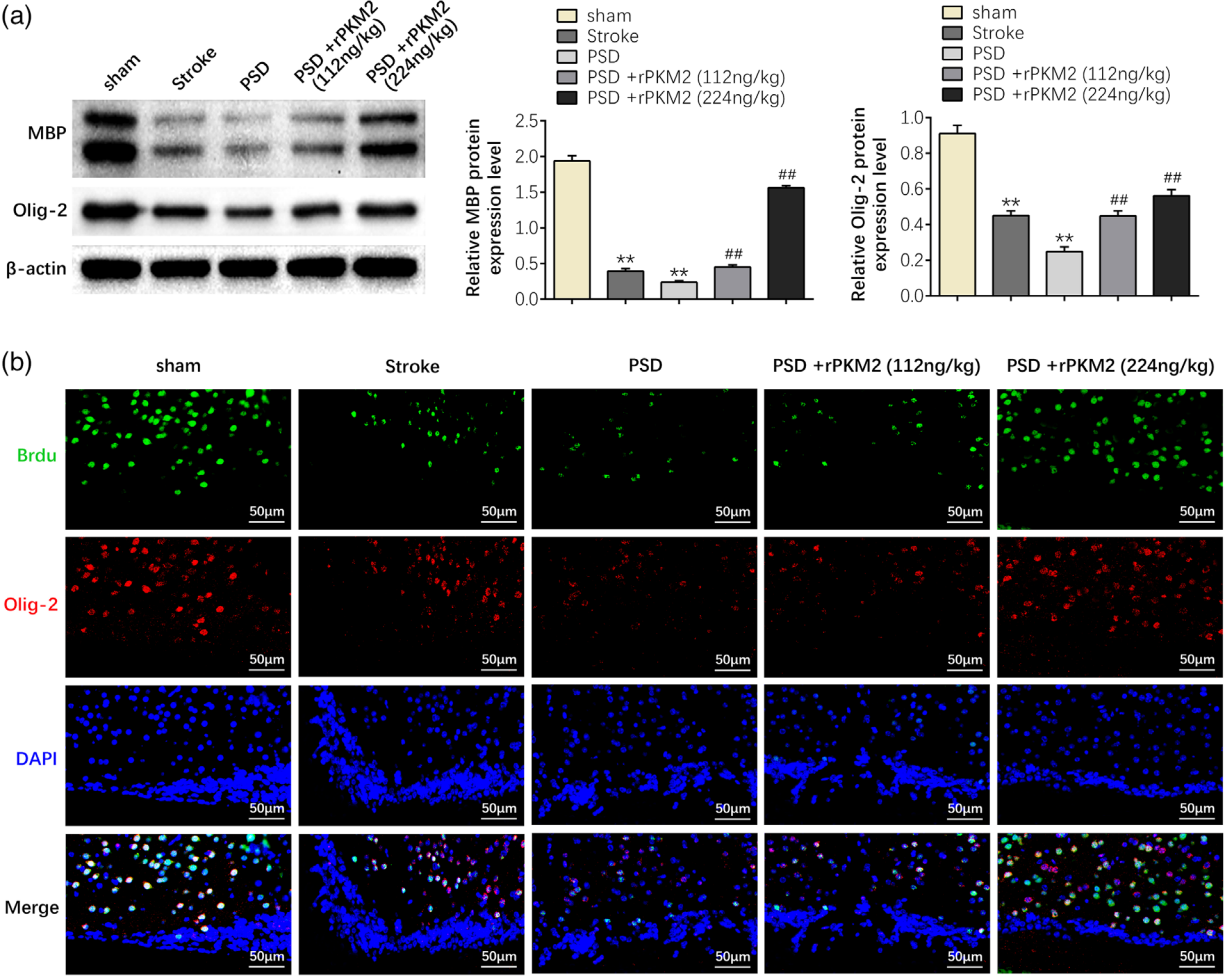
PKM2 can promote the proliferation of oligodendrocytes in PSD rats. (a) The protein expression levels of MBP and Olig‐2 of rats in each group. (b) The number of MBP and Olig‐2 positive oligodendrocytes in PSD rats. Data were presented as the mean ± SD with three independent experiments. **p < .01 versus sham group and ^##^ *p* < .01 versus PSD group

Subsequently, western blot and immunofluorescent staining of subventricular zone (SVZ) of rats in each group were applied to investigate the effect of PKM2 on the proliferation of oligodendrocytes in PSD rats. The results confirmed that compared with the sham group, rat in the stroke or PSD group showed lower levels of Olig‐2, a marker for oligodendrocyte transcription factors, and lower level of MBP, a marker for myelin basic protein, indicating that there were fewer oligodendrocytes and myelin in the SVZ of stroke or PSD rats (*p* < 0.01). However, rats in the PSD group administrated with PKM2 showed increased protein expression level of MBP and Olig‐2 (*p* < 0.01). In addition, the reduction of oligodendrocytes induced by PKM2 changed gradually with increasing dose (*p* < 0.01) (Figure 2a). Furthermore, the proliferation ability of oligodendrocytes administrated with stroke or PSD was reduced, whereas PKM2 restored the proliferation ability of oligodendrocytes administrated with PSD in a dose‐dependent manner (*p* < 0.01) (Figure 2b). These results verified that PKM2 can promote the proliferation of oligodendrocytes in PSD rats.

### 3.3 PKM2 can reduce the inflammatory response in PSD rats

**FIGURE 3:**
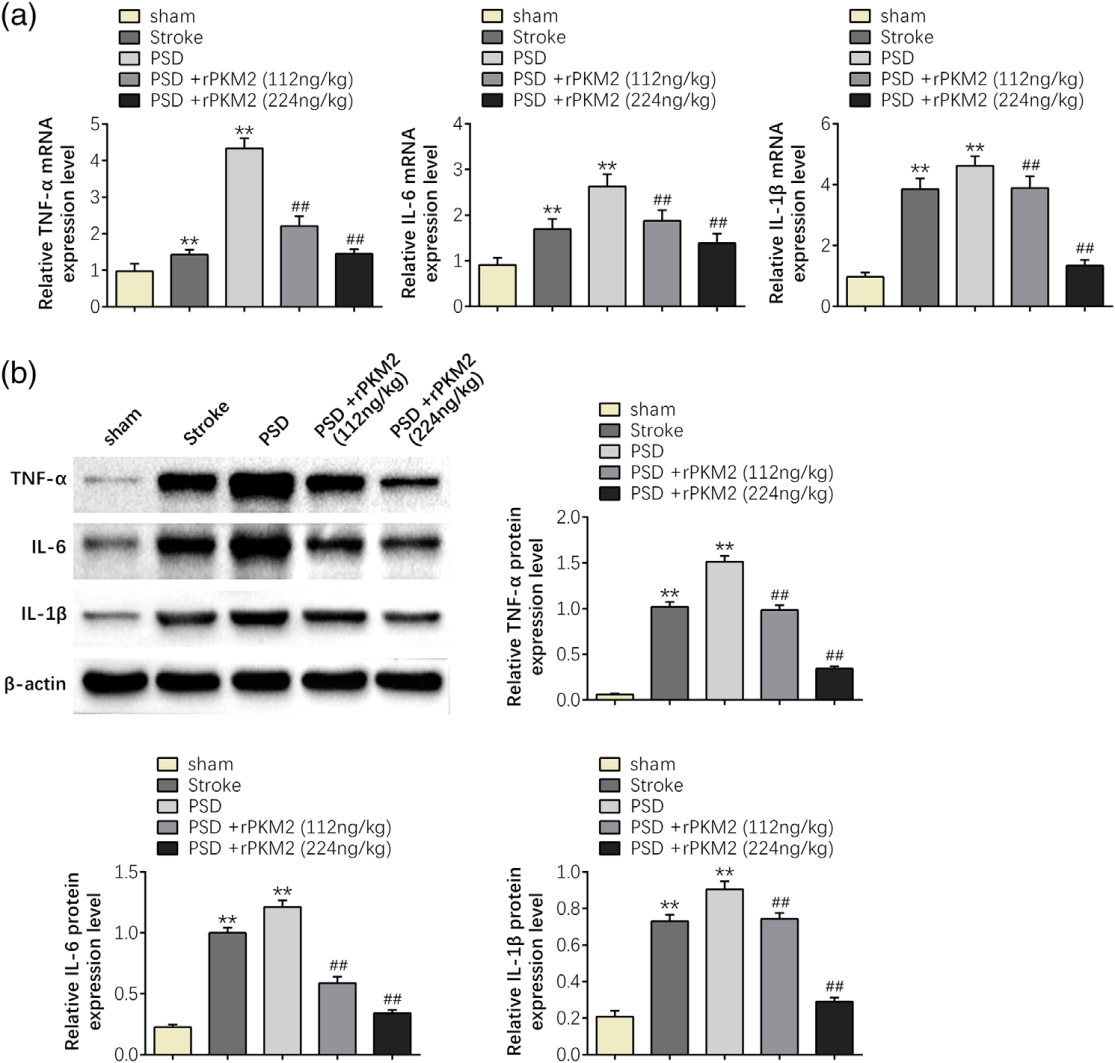
PKM2 can reduce the inflammatory response in PSD rats. (a) The mRNA expression levels of TNF‐α, IL‐6, and IL‐1β of rats in each group. (b) The protein expression levels of TNF‐α, IL‐6, and IL‐1β of rats in each group. Data were presented as the mean ± SD with three independent experiments. **p < .01 versus sham group and ^##^ *p* < .01 versus PSD group

In addition, qPCR and western blot were applied to investigate the mRNA and protein expression levels of TNF‐α, IL‐6, and IL‐1β in serum of rats in each group to ascertain the anti‐inflammatory effect of PKM2 on PSD rats. Both the qPCR and western blot results suggested that compared with the sham group, the mRNA and protein expression levels of TNF‐α, IL‐6, and IL‐1β were strikingly increased in the PSD group (*p* < 0.01), indicating that PSD could induce inflammatory response. However, PKM2 markedly reduced the mRNA and protein expression levels of TNF‐α, IL‐6, and IL‐1β in PSD group (*p* < 0.01). Furthermore, the inflammatory response induced by PSD gradually changed with increasing dose (*p* < 0.01) (Figure 3a,b). These results revealed that PKM2 has a significant anti‐inflammatory effect on PSD rats.

### 3.4 PKM2 can reduce oxidative stress in PSD rats

**FIGURE 4:**
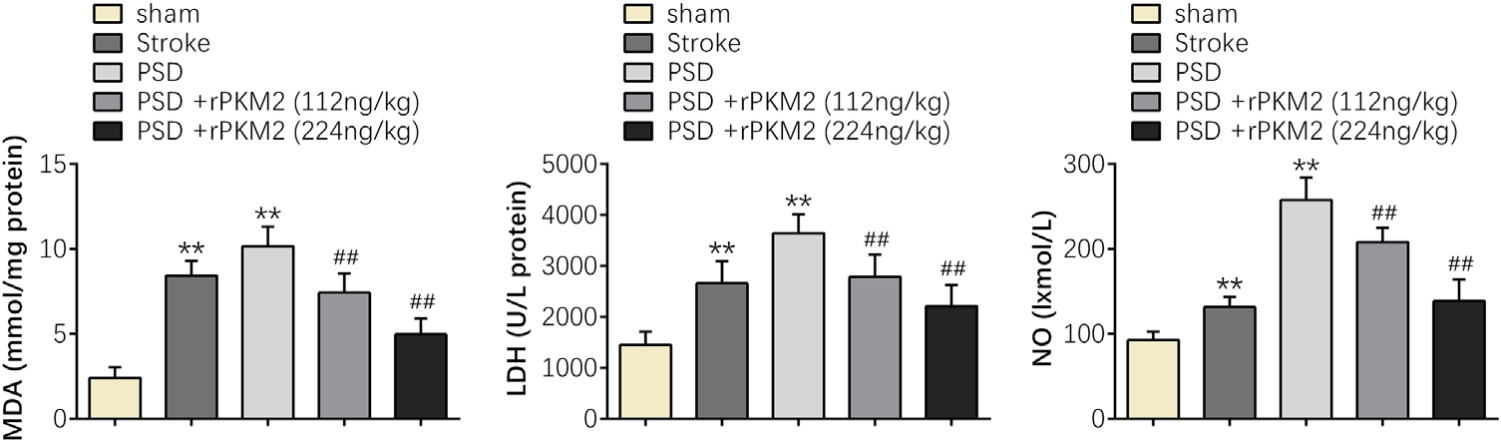
PKM2 can reduce oxidative stress in PSD rats. The protein expression levels of MDA, LDH, and NO of rats in each group. Data were presented as the mean ± SD with three independent experiments. **p < .01 versus sham group and ^##^ *p* < .01 versus PSD group

Furthermore, ELISA was applied to investigate the protein expression levels of malondialdehyde (MDA), lactate dehydrogenase (LDH), and nitric oxide (NO) of rats in each group to ascertain the effect of PKM2 on oxidative stress in PSD rats. The ELISA results hinted that compared with the sham group, the protein expression levels of MDA, LDH, and NO were dramatically increased in the PSD group (*p* < 0.01), indicating that PSD could induce oxidative stress. However, PKM2 obviously reduced the protein expression levels of MDA, LDH, and NO in the PSD group (*p* < 0.01). Furthermore, the oxidative stress induced by PSD gradually changed with increasing dose (*p* < 0.01) (Figure 4). These results proved that PKM2 can reduce oxidative stress in PSD rats.

**FIGURE 5:**
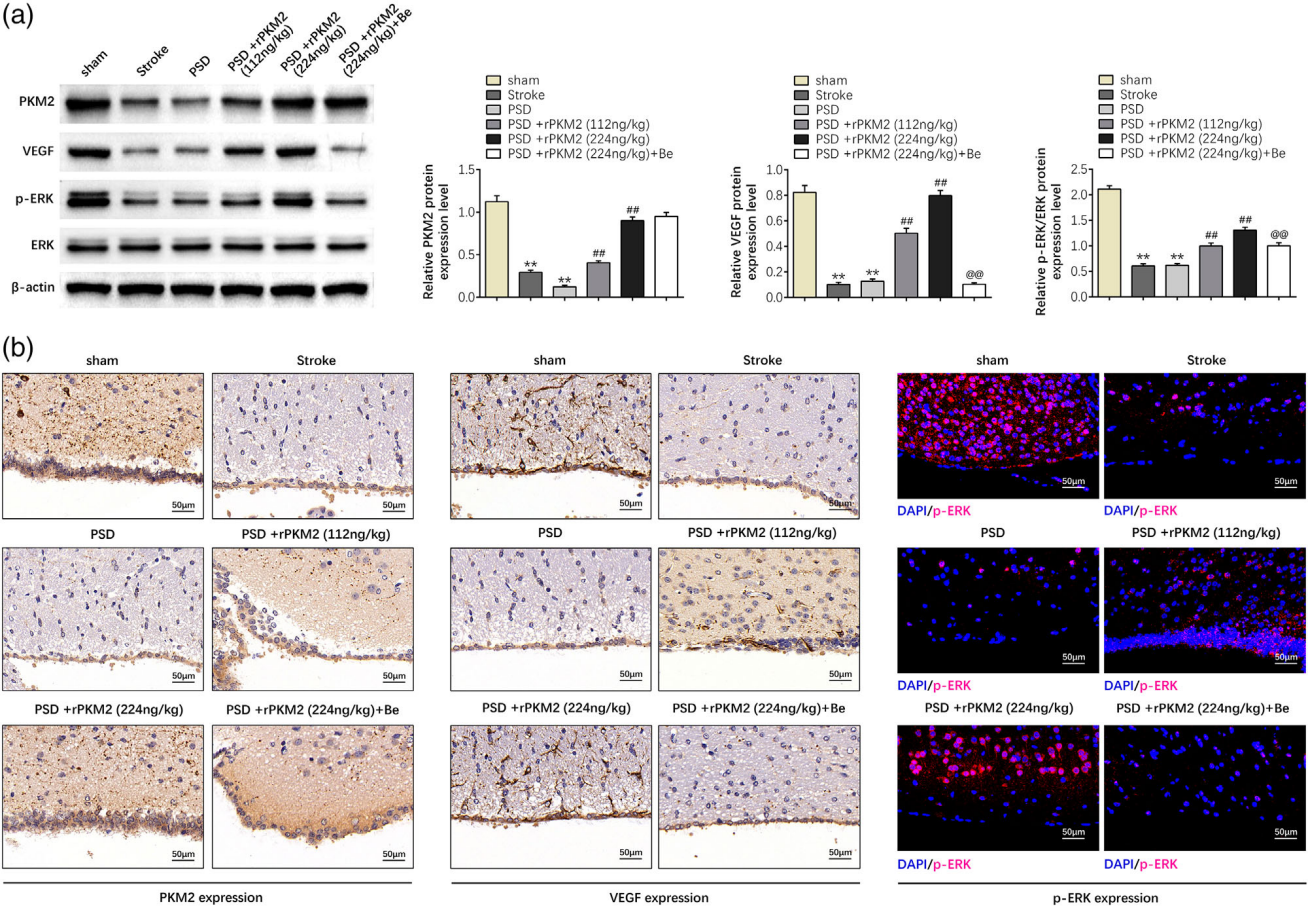
PKM2 promotes activation of the MAPK/ERK pathway by activating VEGF expression. (a) The protein levels of PKM2, ERK, and VEGF in PSD rat, as determined using western blotting. Relatively quantitative results were determined by Image J and shown as histogram. (b) IHC assay was applied to examine the protein levels of PKM2, ERK, and VEGF in PSD rat. Data were presented as the mean ± SD with three independent experiments. **p < .01 versus sham group, ^##^ *p* < .01 versus PSD group, and ^@@^ *p* < .01 versus PSD + rPKM2 (224 ng/kg) group

### 3.5 PKM2 promotes activation of the MAPK/ERK pathway by activating VEGF expression

To estimate that whether PKM2 improves symptoms of post‐ischemic stroke depression in a PSD rat by activation of the MAPK/ERK pathway through activating VEGF expression, western blot, immunohistochemical, and immunofluorescent staining were applied to detect the expression levels of PKM2, ERK, and VEGF in PSD rats. The western blot, immunohistochemical, and immunofluorescent staining results showed that compared with the sham group, the protein expression levels of PKM2, phosphorylated ERK, and VEGF were significantly decreased in the stroke or PSD group (*p* < 0.01). However, exogenous recombinant protein PKM2 obviously enhanced the protein expression levels of PKM2, phosphorylated ERK, and VEGF in the PSD group (*p* < 0.01). Furthermore, the changes of protein expression levels induced by PSD gradually changed with increasing dose (*p* < 0.01). Besides, the protein expression levels of phosphorylated ERK and VEGF were reversibly reduced when PSD rats were administrated with PKM2 and VEGF inhibitor bevacizumab (*p* < 0.01) (Figure 5a,b). These results confirmed that PKM2 promotes the activation of the MAPK/ERK pathway by activating VEGF expression.

## 4 DISCUSSION

Although the medical science has been very developed, the molecular mechanism of post‐ischemic stroke depression remains unclear. Recently, some studies have suggested that pyruvate kinase M2 (PKM2) has the potential to play an important role in anti‐inflammatory (Hu et al., 2018). However, PKM2 is rarely studied in post‐ischemic stroke depression, and the molecular regulatory mechanism of PKM2 in post‐ischemic stroke depression fate determination remains poorly understood. Therefore, understanding the molecular regulatory mechanism of PKM2 in post‐ischemic stroke depression may provide the novel therapeutic targets for post‐ischemic stroke depression.

PKM2 is one of the major cytosolic enzymes which is associated with other glycolytic enzymes, and it possesses various pharmacological properties such as anti‐inflammatory activities (Cheng et al., 2017). Several other studies have reported that the PKM2 markedly improved symptoms of inflammatory lung injury and other diseases(Zhong et al., 2020). Specifically, studies have also found that PKM2 activation may protect against the progression of diabetic (Qi et al., 2017). Recently, the relationship between PKM2 and SIRT5 was confirmed to prevent colitis in mice (Wang et al., 2017). Moreover, PKM2 was found to ameliorate cell proliferation, metabolism, and migration in renal cell carcinoma (Dey et al., 2019). Due to multi‐targeted actions of PKM2, it could have broad prospects in the development of new and safe drugs as a promising cytosolic enzyme. However, few studies had concentrated on the pharmacological value of PKM2 in post‐ischemic stroke depression and its potential mechanism has not been reported yet. This study disclosed that PKM2 can improve the depressive behavior and promote the proliferation of oligodendrocytes in PSD rats. In addition, PKM2 has anti‐inflammatory and anti‐oxidative stress effects on PSD rats. These results suggest that PKM2 may act as a promoting factor in improving symptoms of post‐ischemic stroke depression.

A growing number of studies confirmed that PKM2 exerts its functions by regulating the expression of target mRNAs. A previous study proved that PKM2 promotes lung cancer metastasis via activating the integrin beta1/FAK pathway (Wang et al., 2020). Moreover, PKM2 was reported to inhibit the progression of bladder cancer via MAKP signaling pathway (Zhu et al., 2018). In addition, PKM2 was reported to promote migration, invasion, and EMT of gastric carcinoma by HIF‐1α/BCL‐6 Pathway (Li et al., 2020). Another novel discovery of this study was that ERK and VEGF proteins are the target of PKM2. MAPK/ERK pathway is a protein chain inside cells that carries signal from receptor on the surface of cell to the DNA in the nucleus. It is involved in a variety of cellular processes, including RAS activation, kinase cascade, and regulation of translation and transcription. VEGF is a signal protein produced by cells that stimulates the formation of blood vessels. Here, the fact that PKM2 could target ERK and VEGF was demonstrated by western blot assays. The changes of ERK and VEGF protein expression levels induced by PSD gradually changed with increasing PKM2 dose, which indicates that PKM2 improves symptoms of post‐ischemic stroke depression by activating VEGF to mediate the MAPK/ERK pathway.

In conclusion, in this study, the fact that PKM2 can improve the depressive behavior of PSD rats was discovered. In addition, PKM2 can promote the proliferation of oligodendrocytes in PSD rats. Meanwhile, PKM2 can reduce the inflammatory response in PSD rats. Moreover, PKM2 can reduce oxidative stress in PSD rats. Finally, the fact that PKM2 improve symptoms of post‐ischemic stroke depression by activating VEGF to mediate the MAPK/ERK pathway was also confirmed. All these results figured out the role of PKM2/VEGF/MAPK/ERK signaling pathway in improving symptoms of PSD, which could conceivably pave the path for advanced therapeutic targets in post‐ischemic stroke depression.

## CONFLICT OF INTEREST

The authors state that there are no conflicts of interest to disclose.

## AUTHOR CONTRIBUTIONS

Yun Feng and Xuebin Li designed the study and supervised the data collection. Jie Wang analyzed the data and interpreted the data. Xiaohua Huang, Lanqing Meng, and Jianmin Huang, prepared the manuscript for publication and reviewed the draft of the manuscript. All authors have read and approved the manuscript.

## Data Availability

All data generated or analyzed during this study are included in this published article.
